# Silica bioreplication preserves three-dimensional spheroid structures of human pluripotent stem cells and HepG2 cells

**DOI:** 10.1038/srep13635

**Published:** 2015-09-01

**Authors:** Yan-Ru Lou, Liisa Kanninen, Bryan Kaehr, Jason L. Townson, Johanna Niklander, Riina Harjumäki, C. Jeffrey Brinker, Marjo Yliperttula

**Affiliations:** 1 Centre for Drug Research, Division of Pharmaceutical Biosciences, Faculty of Pharmacy, the University of Helsinki, Helsinki 00014, Finland; 2 Advanced Materials Laboratory, Sandia National Laboratories, Albuquerque, New Mexico 87185, USA; 3 Department of Chemical and Biomolecular Engineering, the University of New Mexico, Albuquerque, New Mexico 87131, USA; 4 Division of Molecular Medicine, Department of Internal Medicine, the University of New Mexico, Albuquerque, New Mexico 87131, USA; 5 Center for Micro-Engineered Materials, the University of New Mexico, Albuquerque, New Mexico 87131, USA

## Abstract

Three-dimensional (3D) cell cultures produce more *in vivo*-like multicellular structures such as spheroids that cannot be obtained in two-dimensional (2D) cell cultures. Thus, they are increasingly employed as models for cancer and drug research, as well as tissue engineering. It has proven challenging to stabilize spheroid architectures for detailed morphological examination. Here we overcome this issue using a silica bioreplication (SBR) process employed on spheroids formed from human pluripotent stem cells (hPSCs) and hepatocellular carcinoma HepG2 cells cultured in the nanofibrillar cellulose (NFC) hydrogel. The cells in the spheroids are more round and tightly interacting with each other than those in 2D cultures, and they develop microvilli-like structures on the cell membranes as seen in 2D cultures. Furthermore, SBR preserves extracellular matrix-like materials and cellular proteins. These findings provide the first evidence of intact hPSC spheroid architectures and similar fine structures to 2D-cultured cells, providing a pathway to enable our understanding of morphogenesis in 3D cultures.

Human pluripotent stem cells (hPSCs), including human embryonic stem cells (hESCs) and induced pluripotent stem cells (hiPSCs), show potential for drug research, tissue engineering, and regenerative medicine. Since the first hESC lines were established in 1998^1^, much research has focused on the development of *in vitro* culture systems to maintain cell pluripotency and to minimize the spontaneous differentiation of hPSCs. For clinical applications, cells cannot come into contact with animal-derived components. One of the reasons is unwanted immune responses^2^. Therefore, a number of synthetic biomaterials^3^,^4^,^5^,^6^,^7^,^8^,^9^ have been produced to replace traditionally used feeder cells and Matrigel as substrata in hESC and hiPSC cultures. However, all of the above-mentioned cultures^3^,^4^,^5^,^6^,^7^,^8^,^9^ are two-dimensional (2D) cell cultures, which do not mimic the *in vivo* three-dimensional (3D) stem cell niche.

To date, there are only a handful of studies demonstrating successful 3D cultures of hPSCs in hydrogels^10^,^11^,^12^,^13^. We recently developed a 3D cell culture system using a plant-derived nanofibrillar cellulose (NFC) hydrogel^14^. The hPSCs form pluripotent 3D spheroids in the NFC hydrogel. The unique feature of the NFC hydrogel-based 3D culture system is that intact 3D spheroids can be recovered from the hydrogel by a cellulase enzyme for downstream applications. We have studied the phenotypic features of the hPSCs in the NFC hydrogel at molecular and functional levels^14^. However, little is known about the detailed cellular morphology and the organization of the cells inside the spheroids. The morphology of the hESCs cultured in 2D environments was previously studied by scanning electron microscopy (SEM), which revealed tight cell-cell contact, microvilli-covered cell surfaces, and matrix-like materials between cells^15^,^16^. By contrast, the morphology of the hPSCs cultured in 3D environments has not been studied in great detail. To our knowledge, there is only one morphological study which showed the spherical shape of the hESCs grown within a porous chitosan-alginate scaffold^11^. To gain insights into the morphology of 3D hPSC spheroids, we employed the silica bioreplication (SBR) method^17^,^18^ to stabilize the spheroids for examination by SEM.

The first *in vitro* biomimetic synthesis of silica was reported more than a decade ago^19^. Later this biomimetic approach was used in producing silica nanomaterials^20^,^21^,^22^ and cell-directed silica biocomposites^17^,^23^,^24^,^25^,^26^,^27^. SBR is a self-limiting biomolecular surface-directed silica assembly process that results in nearly an exact replica of external and internal cellular^17^,^27^, tissue, and organism-scale^18^ features in nanometre (<10 nm) thick silica layers. Specimens are incubated in a dilute (100 mM) solution of silicic acid (Si(OH)_4_) that is mildly acidic to suppress self-condensation of silica precursors (≡Si-OH + HO-Si≡ → ≡Si-O-Si≡ + H_2_O) which would lead to bulk gel formation. Only in close proximity to proteinaceous biomolecular surfaces, which serve as silica condensation catalysts, does silica deposition occur. Once the catalytic sites are occluded, deposition is terminated, resulting in precise replication of biomolecular features. Silica replication causes the entirety of hierarchical features displayed by multicellular structures to be mechanically stabilized allowing simple drying of the specimen without significant dimensional changes.

In this study, we looked at the structures of the cells in 3D spheroids and 2D surfaces after SBR. Moreover, we show that molecular-scale antigen presentation is preserved under SBR conditions.

## Results

### The phenotypic features of the cells in 2D and 3D cultures

**Figure 1:**
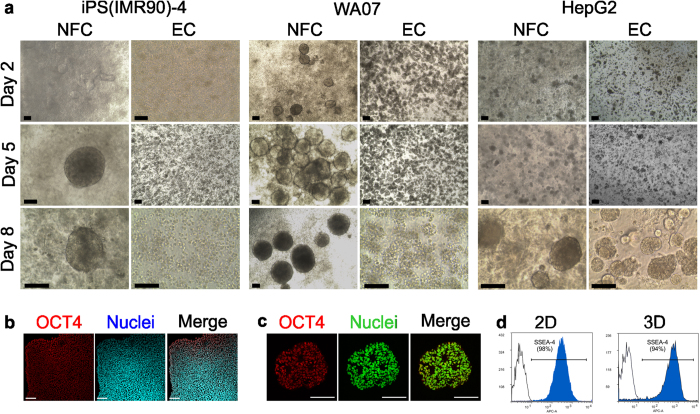
The morphology of hiPSCs iPS(IMR90)-4, hESCs WA07, and human hepatocellular carcinoma HepG2 cells cultured in 3D hydrogels and the pluripotency of WA07 cells. (**a**) WA07 and iPS(IMR90)-4 cell spheroids in the NFC hydrogel (NFC) but not in the ExtraCel™ hydrogel (EC). HepG2 cell spheroids in both NFC and EC hydrogels. Images are representative of eight biological samples from NFC hydrogels and three biological samples from EC hydrogels. (**b**,**c**) Immunostaining of the pluripotency marker OCT4 in WA07 cells cultured in standard 2D culture system (**b**) and in the NFC hydrogel for 7 days (5 μm paraffin section) (**c**). (**d**) Flow cytometry analysis of the pluripotency marker SSEA-4 in WA07 cells after being cultured in 2D and in 3D NFC hydrogel for 7 days. Scale bars = 100 μm.

We cultured both the hPSCs and HepG2 cells in the NFC hydrogel, which has recently been shown to be a suitable hydrogel for 3D cell culturing^14^,^28^,^29^, and in the ExtraCel™ hydrogel, a hyaluronan-gelatine-based hydrogel. Phase contrast microscopy images reveal that both iPS(IMR90)-4 and WA07 cells form round 3D spheroids with diameters between 100 μm to 350 μm during 8-day culture in the NFC hydrogel, but not in the ExtraCel™ hydrogel (Fig. 1a). We observed a large degree of variation in the sizes of individual WA07 spheroids, which is expected given that they are formed from individual colonies containing a variable number of stem cells. Indeed, the number of cells counted (via dissociation into individual cells) from three individual spheroids showed a wide range (1056–6720 cells). The cell viability estimated by trypan blue exclusion is over 97%. The pluripotent markers of hPSCs were studied by immunofluorescence and flow cytometry. WA07 cells expressed the pluripotent markers OCT4 and SSEA-4 at similar levels in both the standard 2D culture and 3D NFC hydrogel culture (Fig. 1b–d). HepG2 cells formed 3D spheroids on day 8 with diameters at 73 ± 21 μm (n = 71) in the NFC hydrogel and 66 ± 19 μm (n = 47) in the ExtraCel™ hydrogel, respectively.

### SBR preserves detailed cellular structures

**Figure 2:**
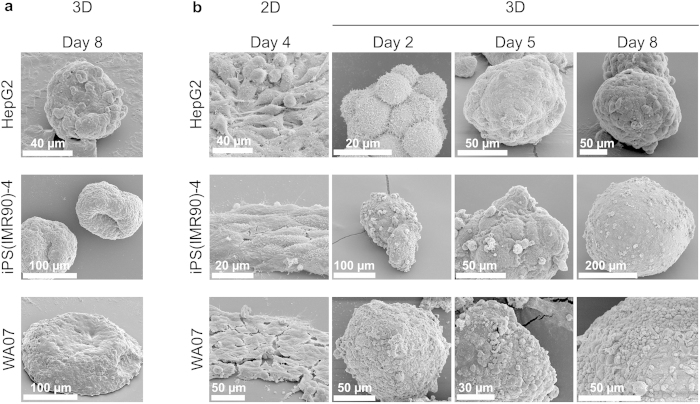
Morphology of human pluripotent stem cells and hepatocellular carcinoma cells with and without silica bioreplication. (**a**) SEM images of HepG2, iPS(IMR90)-4, and WA07 cell spheroids show deformation of 3D spheroids. (**b**) SEM images of HepG2, iPS(IMR90)-4, and WA07 cell spheroids after silica bioreplication show well-preserved spheroid architecture and tight cell-cell contact in the NFC hydrogel-based 3D cultures. Images are representative of eight biological samples.

To study the detailed cellular structures of the cells cultured in 2D and 3D, we prepared the cell samples and cell-silica composites for SEM. We observed dramatic differences between non-silicified spheroids and silicified spheroids. The non-silicified spheroids deformed considerably during sample preparation, presumably during the drying procedure, resulting in obscuration of surface features (Fig. 2a). In contrast, the cell spheroid-silica composites stabilized using SBR retained their spherical morphology and were well preserved (Fig. 2b). Both the hPSCs and HepG2 cells developed tight cell-cell interactions during 8-day 3D culture in the NFC hydrogel (Fig. 2b). Cells in 3D spheroids appeared more round than those in 2D culture (Fig. 2b). We observed protrusions in some elongated HepG2 cells and also in some hPSCs at the edge of the colonies, but not in the cells in 3D spheroids (Fig. 2b). Some small cracks were observed in the hPSC colonies in 2D culture (Fig. 2b), which was presumably caused by dehydration during sample preparation for SEM.

For comparison with the NFC hydrogel-based 3D culture, we obtained HepG2 cell spheroids in the ExtraCel™ hydrogel. However, we were not able to recover the intact HepG2 cell spheroids from the ExtraCel™ hydrogel because the enzyme mixture used to degrade the hydrogel was observed to dissociate the cell spheroids into single cells. Therefore, we could only study the silicified HepG2 cell spheroids inside the ExtraCel™ hydrogel; however, the SEM images provide little information on the cells due to the presence of the hydrogel (Supplementary Fig. 1).

**Figure 3:**
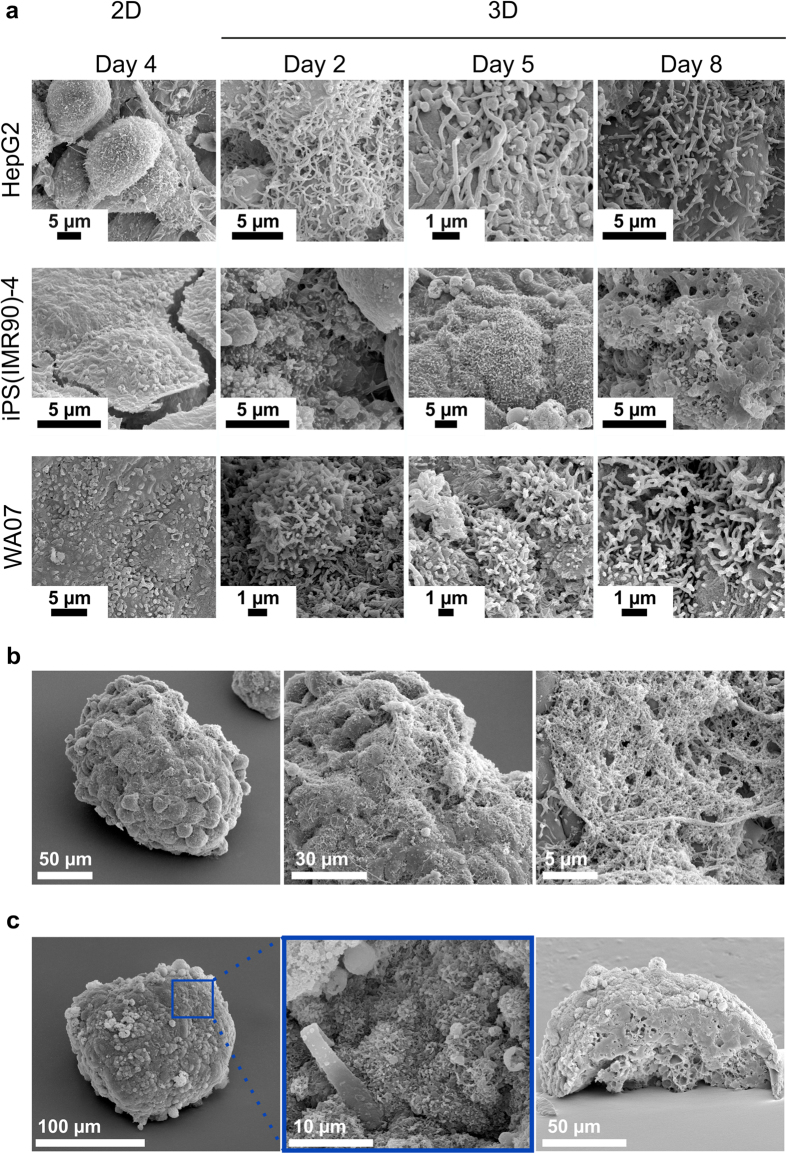
Scanning electron microscopy of silicified cells. (**a**) Microvilli-like structures on the surface of HepG2, iPS(IMR90)-4, and WA07 cells in 2D culture and in 3D NFC hydrogel culture. (**b**) Extracellular matrix-like material on a HepG2 cell spheroid in the NFC hydrogel for 8 days. (**c**) Silica-replicas of WA07 spheroids (5 days in the NFC hydrogel) after calcination. Images are representative of eight biological samples.

At higher magnification we observed fine cellular structures on the cell membrane of the cell-silica composites. Dense microvilli-like structures were observed in the hPSCs and HepG2 cells cultured in both 2D and 3D (Fig. 3a). Such structures were observed on the surface of hESCs in an earlier study^15^. Surprisingly, we observed abundant extracellular materials on the surfaces of HepG2 cells cultured in the NFC hydrogel (Fig. 3b). These extracellular materials are likely produced by HepG2 cells since the NFC hydrogel has been degraded by cellulase and the silicified nanofiber bundles of the NFC hydrogel appear different (Supplementary Fig. 2).

We removed organic cellular materials from WA07 spheroid-silica composites via calcination (500C in air for 16–24 hrs) and produced 3D silica-replicas of WA07 spheroids with well-preserved spheroid architectures and cell surface structures (Fig. 3c). The cross-sectional image of a quarter calcined spheroid shows that the interior was completely silicified with some cavities near the centre of the spheroid (Fig. 3c).

### Cellular antigens are partially preserved during SBR

**Figure 4:**
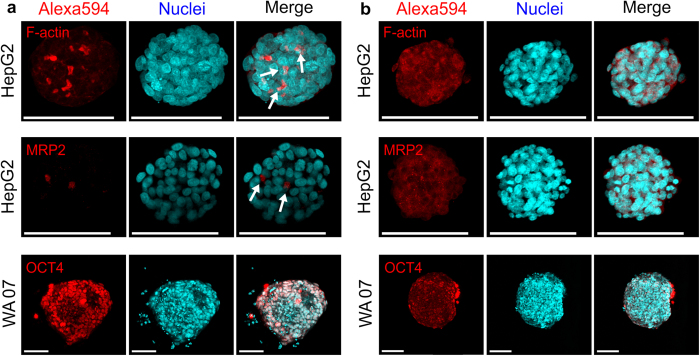
Detection of cellular proteins in desilicified cells. (**a**) Staining of filamentous actin (F-actin) and multidrug resistance-associated protein 2 (MRP2) in HepG2 spheroids after 8 day-culture in the NFC hydrogel. Immunostaining of the pluripotency marker OCT4 in WA07 cells after 5 day-culture in the NFC hydrogel. (**b**) Detection of F-actin, MRP2, and OCT4 after desilicification of spheroid-silica composites. Scale bars = 100 μm. Images are representative of eight biological samples.

Cellular antigens are commonly used as a means of identifying and characterizing specific cell populations. As such, here we examined the extent to which specific cellular antigens are preserved during SBR by immunofluorescence. Before immunofluorescence imaging, silica was etched from spheroid-silica composites by a dilute, buffered hydrofluoric acid. The resulting spheroids were stained by either an F-actin probe or specific antibodies. HepG2 cells cultured in the NFC hydrogel formed multiple bile canaliculus-like structures, which were revealed by the polarised distribution of F-actin and apical localisation of the multidrug resistance-associated protein 2 (MRP2) (Fig. 4a). After SBR, MRP2 antigens in HepG2 cells were still detectable by immunofluorescence. However, the antigens had diffused throughout the cytoplasm and even spread to other cells in the spheroids, as the protein was not detected at the apical domain of the cell membrane (Fig. 4b). Similarly, the polarised distribution of F-actin was lost during SBR, though it was still detectable in the cytoplasm (Fig. 4b). In contrast, a stem cell marker, OCT4, a nuclear protein, was detected in the cell nuclei of WA07 spheroids cultured in the NFC hydrogel both before and after SBR (Fig. 4). Negative controls in immunofluorescence show no positive signal (Supplementary Fig. 3).

## Discussion

The use of various 3D culture systems has become an increasingly common method of cell culture in different areas, including cancer research, drug research, and tissue engineering. Studying the cell morphology and spatial organization within a 3D environment helps better understand how cells migrate and organize into defined patterns during tissue formation, which is an important part of developmental biology and cancer biology, and this kind of studies could potentially lead to improved methods of regulating cellular behaviour. In the present study, we showed the formation of 3D spheroids of hPSCs and HepG2 cells in the NFC hydrogel. By using the SBR method we were able to retain the spheroid architecture and reveal the detailed cell membrane features and cell organization within the spheroids at various stages of spheroid development. The hPSCs and HepG2 cells in the spheroids are more round than those in 2D cultures, and they develop tight cell-cell interactions and microvilli-like structure-coated membranes as seen in 2D cultures. Such microvilli-like structures were earlier observed on the surface of hESCs cultured on the surface of microcarriers^15^. To our knowledge, no such microvilli-like structures have been shown on 3D hESC spheroids by SEM^11^. Furthermore, the HepG2 cells cultured in the NFC hydrogel produced a greater amount of extracellular matrix-like materials than those in 2D culture. The HepG2 cell spheroids observed under SEM appear similar to those reported earlier^30^,^31^. Extracellular matrix was previously observed on the HepG2 cell spheroids under SEM, but there was no comparison with 2D cultured cells^30^. These findings presented here provide detailed visual evidence of the hPSC and HepG2 cell spheroid architecture, and will enable further studies of morphogenesis in 3D cultures.

In contrast to the handful of biomaterial-based 3D culture systems for hPSCs, the NFC hydrogel-based 3D culture used here enables the recovery of intact 3D cell spheroids, allowing ready compatibility with various downstream applications and analyses. In comparison with the NFC hydrogel, the intact spheroids cannot be recovered from the ExtraCel™ hydrogel. Cell spheroids can also be generated in biomaterial-free culture systems, such as suspension culture and hanging drops. Best to our knowledge, hPSCs have been cultured in suspension, but not in hanging drop system. Karyotypic abnormalities and necrosis were found in hPSC aggregated in suspension culture^32^,^33^. HepG2 spheroids were previously generated in hanging drops^34^.

Earlier studies on hESC spheroids^11^ and hESC-derived hepatocyte spheroids^35^ using SEM showed very little subcellular information on dehydrated samples. Though SEM of SBR spheroids does not approach the level of detail that could be attained using thin sectioning and imaging via transmission electron microscopy (TEM), it leaves the global structure intact, and potentially addressable using other 3D imaging techniques such as confocal microscopy, as shown in Fig. 4. This compatibility with fluorescence imaging should prove enabling for colocalisation studies that can employ advanced fluorescence imaging (e.g., super resolution microscopy) provided that structures of interest (for post labelling) and/or fluorophores (for pre-labelling) can survive the SBR process—a question to be addressed in future work.

We found that SBR proved essential for obtaining intact 3D architectures of cell spheroids observable using SEM. Silica serves as a supporting scaffold in maintaining the shape of spheroids during the dehydration procedure. To compare sample stability in the absence of silica treatment, we prepared spheroids for SEM imaging using a well-established serial dehydration approach and drying from HMDS (see methods for details). This sample preparation method has been shown to preserve cellular features as well as critical point drying^36^. However, we observed substantial structural deformation/collapse following drying and significant obscuration of surface features compared to silica stabilised spheroids (Fig. 2a). SBR has previously been used to create complex biomaterials with hierarchical features^22^,^37^ and to preserve single cell suspension and attached cells^17^,^27^. To our knowledge, this is the first time that SBR is used to preserve the 3D architecture of cell spheroids. This enables us to study the spheroids at high resolution and broaden our knowledge on hPSCs cultured in 3D, as has recently been demonstrated at the tissue, organ, and organism levels. This would be a convenient way of preserving biological samples and creating novel biomaterials, as suggested earlier^38^. It may be possible to study silicified spheroids by TEM, cryo-TEM, and super resolution microscopy to obtain more detailed information on the cellular structures. In this study, we chose SEM because it provides rapid analysis of the global architecture of a biological sample versus the arduous 2D sectioning necessary for TEM analysis. Indeed using cryo-TEM it should be possible to study the cellular structures in greater detail. However, this technique remains highly specialized involving preparing micro-slice samples (100 micrometer) for TEM and cryo-TEM and virtual reconstruction into a 3D picture. In addition, thin sectioning for cross-sectional imaging in TEM destroys the 3D structures of cell spheroids.

We also discovered that the nuclear protein OCT4 was well preserved during SBR (Fig. 4). However, the localization of a cell membrane-bound protein MRP2 and F-actin was lost during SBR. We postulate that the cell membrane might be disrupted during silica deposition, and subsequently the membrane-bound antigen and F-actin diffused throughout the cytoplasm. Indeed, delocalization of membrane lipid following silica deposition was reported earlier^17^.

In conclusion, by using SBR, we demonstrate here that hPSCs and HepG2 cells cultured in the 3D NFC hydrogel display similar cellular features to those cultured in 2D. The spheroid shape and nuclear antigen was preserved during SBR, which enables sample analyses by different methods including SEM and immunofluorescence.

## Methods

### Cell maintenance

The hESC line WA07^1^ and iPSC line iPS(IMR90)-4^39^ were purchased from WiCell. Stem cells were maintained on Matrigel-coated 6-well plates in mTeSR™1 medium (05850, STEMCELL™ Technologies) which was changed daily. Matrigel coatings were produced by incubating Matrigel (Matrigel basement membrane matrix growth factor reduced, BD Biociences, 356230) dilution (0.5 mg per one 6-well plate) in wells for one hour at room temperature. Stem cells were passaged at a ratio of 1:6 every four days after manual removal of differentiated cells. Versene 1:5000 (Invitrogen, 15040033) was used to detach the stem cell colonies. The human hepatocellular carcinoma HepG2 cells from ATCC (HB-8065) were maintained in 75 cm^2^-cell culture flasks in DMEM with high glucose, GlutaMAX™, and pyruvate (Gibco, 31966) supplemented with 10% fetal bovine serum, 100 U/ml penicillin, and 100 ug/ml streptomycin. The medium was renewed three times per week. HepG2 cells were passaged at a ratio of 1:6 every 3–4 days using TrypLE™ Express (Gibco, 12604-021). All cell cultures were maintained at 37 °C in a humid atmosphere with 5% CO_2_.

### 2D and 3D cell cultures

Before SBR, all the cells were cultured in standard 2D culture and in 3D culture using either the NFC hydrogel (GrowDex™, UPM-Kymmene, Espoo, Finland) or a commercial ExtraCel™ (EC) hydrogel, a hyaluronan-gelatin based hydrogel (Glycosan biosystems, GS208). 2D cultures were performed in 35 mm glass bottom dishes (MatTek Corporation, P35G-1.510-C). For 2D cultures, stem cells were passaged at a ratio of 1:6 on Matrigel coating as described above. The seeding density for HepG2 cells was 40,000 cells/cm^2^. 3D cultures were performed in 8-well Lab-Tek® Chamber Slide™ systems (Nunc, 177445). 3D cultures of stem cells and HepG2 cells in the NFC hydrogel were performed as described earlier by us^14^,^28^. Briefly, the detached stem cell colonies or HepG2 cells were mixed with 0.5 w.t% or 1.0 wt.% NFC hydrogel, respectively. The EC hydrogel formation and cell encapsulation were performed according to the manufacturer’s protocol. An equal medium volume to hydrogel volume was added on top of the NFC and EC hydrogels. The stem cell colony density was five times higher than that in 2D cultures, and the HepG2 cell density in the hydrogels was 1 × 10^6^ cells/ml. The media were renewed daily for all the stem cell cultures and every 3–4 days for all the HepG2 cell cultures. To count cell number in hPSC spheroids, WA07 spheroids were dissociated by Trypsin, and then the single cells were counted by trypan blue exclusion.

### Enzymatic removal of the hydrogels

The NFC hydrogel was degraded with a cellulase enzyme (VTT, Turku, Finland) and the EC hydrogel with 1 × collagenase/hyalurodinase (StemCell Technologies, 07912). Cellulase treatment was performed as described earlier by us^14^. Briefly, 300 μg of cellulase per 1 mg NFC was incubated for 24 hours at 38 °C on a shaker. Spheroids were subsequently washed with 1 × DPBS(-) to remove cellulase enzyme. The EC hydrogel was removed according to the manufacturer’s instructions. However, intact spheroids could not be recovered from the EC hydrogel; instead, enzymatic digestion resulted in single cells.

### Flow cytometry

3D WA07 spheroids were first recovered from the NFC hydrogel with cellulase enzyme as described above. Next, the spheroids were disintegrated to single cells with a Cell Dissociation Buffer (Gibco, 13151-014) followed by Accutase (Merck Millipore, SCR005). The cells were first incubated with anti-SSEA-4 (Developmental Studies Hybridoma Bank, MC-813-70, 1:400 in 2% FBS) on ice for 60 min. After washing, the cells were incubated with goat anti-mouse IgG (H + L), conjugated with APC (SouthernBiotech, 1031-11S, 1:300 in 2% FBS) on ice for 40 min. The negative control sample was stained with only the secondary antibody. The cells were analysed on a BD LSR II flow cytometer (633 nm laser, 660/20 BP filter detector) using BD FACSDiva software. The overlay histograms were created with FlowLogic software.

### Silica bioreplication (SBR) and silica replica fabrication by calcination

Prior to SBR, 2D cultured cells and recovered 3D spheroids were fixed. Both stem cells and HepG2 cells were fixed with 4% paraformaldehyde (PFA) for 10 min after four days in 2D cultures, 15 min (3D HepG2 spheroids), or 30 min (3D stem cell spheroids) after two, five, and eight days in 3D cultures. SBR of 2D cultured cells was performed as described previously^17^. Spheroids were incubated in a 100 mM tetraethyl orthosilicate (TMOS) solution in 1 mM HCl at 38 °C for 24–72 hours on a shaker. HepG2 3D spheroids in the intact EC hydrogel culture were silicified in Lab-Tek® Chamber Slide™ systems in the TMOS solution at 38 °C for 72 hours. Silicified cells were sequentially washed with nano-pure water at pH 3, 1:1 water-methanol solution, and finally 100% methanol and subsequently dried in air. To fabricate 3D silica replica without organic material, 3D WA07 spheroid-silica composites were calcined in air at 500 °C for 16–24 hrs.

### Fixation and dehydration of non-silicified spheroids

Samples were fixed in 4% PFA in PBS for 2 hrs followed by rinsing in PBS and H_2_O. Samples were subsequently dehydrated using 10-min duration sequential washes of 1:10, 1:2, 1:1: 2:1 (ethanol:water), 100% ethanol (2X), 1:1 (ethanol:hexamethyldisilazane [HMDS]), 100% HMDS, and dried overnight in air.

### Scanning electron microscopy

Samples were deposited onto either borosilicate cover glasses or silicon substrates and sputter-coated with Au/Pd to an approximate thickness of 10 nm. SEM images were recorded using an FEI Quanta series scanning electron microscope.

### Immunofluorescence and immunohistochemistry

Silicified hPSC and HepG2 3D spheroids were first treated with a dilute, buffered hydrofluoric acid (Transene, TIMETCH) to remove silica. The spheroids were incubated with the acid for 4 minutes and washed extensively with 1xDPBS(-). The resulting desilicified 3D stem cell and HepG2 spheroids were permeabilised with 0.1% Triton X-100 for 30 minutes. After overnight blocking with normal goat serum (Millipore), spheroids were incubated with either anti-Oct-3/4 (Santa Cruz Biotechnology, sc-9081, 1:500) or anti-MRP2 (abcam, ab3373, 1:50), and negative control spheroids with either rabbit IgG (Santa Cruz Biotechnology, sc-2027, 1:1000) or mouse IgG (Santa Cruz Biotechnology, sc-2025, 1:80) overnight at 4 °C. The secondary antibody either anti-rabbit or anti-mouse conjugated with Alexa Fluor 594 (Invitrogen, 1:400) was incubated for 5 hours at room temperature. Filamentous actin (F-actin) was stained with Alexa Fluor 594 phalloidin (Invitrogen, A12381, 1:50) overnight. Nuclei were stained with either DAPI (Sigma, D8417) for 5 minutes or SYTOX Green (Invitrogen; S7020) for 30 minutes. For confocal imaging, spheroids were placed in a black glass bottom optical imagining 96-well microplate (Matrical Bioscience, MGB096-1-2-L-G-L) and mounted with either ProLong® Gold antifade reagent (Invitrogen, P36934) or SlowFade® Gold antifade reagent (Invitrogen, S36937). The samples were analysed with a Leica TCS SP5II HCS A confocal microscope using UV for DAPI, Argon 488 nm laser for SYTOX Green, and DPSS 561 nm laser for Alexa Fluor 594. The slice displays or 3D projections of the confocal images were created with Imaris software (Bitplane AG).

To analyse the cells inside the spheroids, histological paraffin sections were generated. After fixed in 4% PFA, the spheroids were embedded in HistoGel (Thermo Scientific) and thereafter in paraffin. Five-micrometre thick sections were cut at the Finnish Centre for Laboratory Animal Pathology and used for immunohistochemistry.

## Additional Information

**How to cite this article**: Lou, Y.-R. *et al.* Silica bioreplication preserves three-dimensional spheroid structures of human pluripotent stem cells and HepG2 cells. *Sci. Rep.* **5**, 13635; doi: 10.1038/srep13635 (2015).

## Supplementary Material

Supplementary Information
