# Supplementary material for: Silica bioreplication preserves three-dimensional spheroid structures of human pluripotent stem cells and HepG2 cells
Source: Sci Rep. 2015 Sep 1;5:13635. doi: 10.1038/srep13635 (PMC4555166; doi:10.1038/srep13635)
Supplement: Supplementary Information [file srep13635-s1.pdf]

Silica bioreplication preserves three-dimensional spheroid structures of human pluripotent stem cells and HepG2 cells

Yan-Ru Lou[1,#,*], Liisa Kanninen[1,#], Bryan Kaehr[2,3], Jason L. Townson[4,5], Johanna Niklander[1], Riina Harjumäki[1], C. Jeffrey Brinker[2,3], Marjo Yliperttula[1,*]

[1] Centre for Drug Research, Division of Pharmaceutical Biosciences, Faculty of Pharmacy, the University of Helsinki, Helsinki 00014, Finland

[2] Advanced Materials Laboratory, Sandia National Laboratories, Albuquerque, New Mexico 87185, USA

[3] Department of Chemical and Biomolecular Engineering, the University of New Mexico, Albuquerque, New Mexico 87131, USA

[4] Division of Molecular Medicine, Department of Internal Medicine, the University of New Mexico, Albuquerque, New Mexico 87131, USA

[5] Center for Micro-Engineered Materials, the University of New Mexico, Albuquerque, New Mexico 87131, USA

# These authors contributed equally to this work.

* Correspondence and requests for materials should be addressed to Y.-R.L. or M.Y. (emails: yan-ru.lou@helsinki.fi; marjo.yliperttula@helsinki.fi)

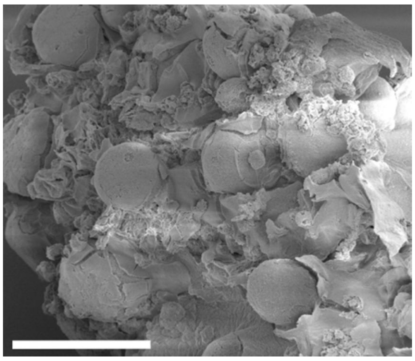

Supplementary Figure 1 Human hepatocellular carcinoma HepG2 cells in the Extracel™ hydrogel (EC). Whole 3D spheroids could not be released from the EC hydrogel and thus detailed cellular structures cannot be visualised due to the presence of the biomaterial scaffold. Scale bar = 100 µm.

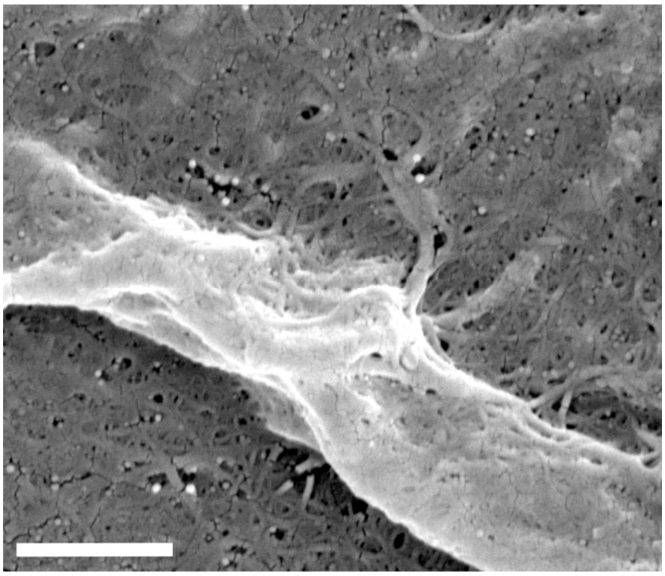

Supplementary Figure 2 Silicification preserves nanoscale structures of the NFC hydrogel. SBR of the NFC hydrogel was performed in the same way as for cell spheroids, as described in the Methods. Scale bar = 1 µm.

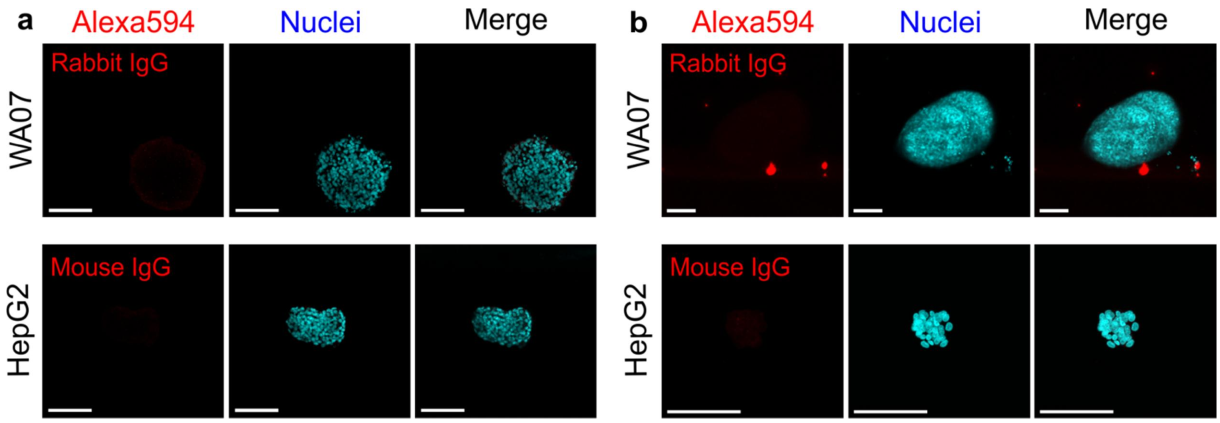

Supplementary Figure 3 Negative controls in immunofluorescence. Stainings of rabbit IgG and mouse IgG controls in WA07 and HepG2 spheroids (**a**) after silicification and desilicification. (**b**) without silicification. Scale bars = 100 µm.
